# Miniaturized 3D-Printed Cell Enables Water/Ethanol Quantification Using Electrochemical Impedance Spectroscopy

**DOI:** 10.3390/s24010131

**Published:** 2023-12-26

**Authors:** Pablo A. Paixao, Flávio S. Michels, Samuel L. Oliveira, Alem-Mar B. Goncalves, Cauê A. Martins, Anderson R. L. Caires, Diego C. B. Alves

**Affiliations:** 1 Applied Nanomaterials and Devices, Institute of Physics, Federal University of Mato Grosso do Sul, Campo Grande 79070-900, MS, Brazilflavio.michels@ufms.br (F.S.M.); alem-mar.goncalves@ufms.br (A.-M.B.G.); 2 Optics and Photonics Group, Institute of Physics, Federal University of Mato Grosso do Sul, Campo Grande 79070-900, MS, Brazil; samuel.oliveira@ufms.br (S.L.O.); anderson.caires@ufms.br (A.R.L.C.); 3 Electrochemistry Research Group, Institute of Physics, Federal University of Mato Grosso do Sul, Campo Grande 79070-900, MS, Brazil; caue.martins@ufms.br

**Keywords:** ethanol content, fuel, 3D-printed, impedance spectroscopy

## Abstract

A miniaturized and low-cost electrochemical 3D-printed system for rapid and accurate quantification of ethanol content in ethanol fuel using electrochemical impedance spectroscopy (EIS) was developed. The monolithic design of the system incorporates insulating thermoplastic electrode separators, with only the cover being mobile, allowing for easy assembly and handling. The portable device, measuring approximately 26 × 24 mm, has a maximum capacity of 1 mL, making it suitable for lab-on-a-chip and portable analysis. By utilizing the dielectric constant of ethanol and ethanol fuel mixtures with water, the miniaturized EIS cell quantifies ethanol content effectively. To validate its performance, we compared measurements from four gas stations with a digital densimeter, and the values obtained from the proposed system matched perfectly. Our miniaturized and low-cost electrochemical 3D-printed device can be printed and assembled in two hours, offering a cost-effective solution for fast and precise ethanol quantification. Its versatility, affordability, and compatibility with lab-on-a-chip platforms make it easily applicable, including for fuel quality control and on-site analysis in remote locations.

## 1. Introduction

Renewable energy production has gained significant importance owing to its potential to mitigate social and environmental impacts, decrease air pollution, and augment the overall quality of life. As a result, biofuels have emerged as a sustainable substitute for fossil fuels, providing an eco-friendly alternative. Bioethanol, obtained through fermentation of biomass sources, including sugarcane and corn, exhibits low pollutant emissions and can potentially replace conventional fuels derived from oil and natural gas [1]. For instance, bioethanol is popular in Brazil due to its cost-effectiveness and reduced environmental impact when used in automotive engines [2,3]. In 2022, more than 590 million tons of sugarcane were processed to produce 58 billion liters of bioethanol [4,5].

Bioethanol has two grades of classification in automotive engines: anhydrous ethanol (AE) when blended with gasoline and hydrous ethanol (HE) when used solely as a fuel [6]. The bioethanol adulterated with water damages vehicles by accelerating corrosion and wear of engine parts due to the release of an unusual amount of water for an extended period [7]. Thus, water-adulterated bioethanol is a common issue that national regulatory agencies must address, especially for HE, due to their high miscibility and similar coloration. Therefore, it is crucial to implement rigorous procedures for the quality control of bioethanol to ensure environmental benefits and engine maintenance.

The National Petroleum, Natural Gas and Biofuels Agency (ANP) of Brazil regulates a maximum limit of 4.5% (*v*/*v*) or 7.5% (*w*/*w*) of water in HE [7]. There are three standard methods to quantify water in ethanol to assure quality in HE: (i) digital densimeter by quantifying the weight of ethanol; (ii) gas chromatography (GC) by determining the ethanol volume; and (iii) Karl Fischer method by obtaining the mass of water [7]. Despite being practical, the existing methods show some drawbacks. The densimeter is low-cost and straightforward but is not very accurate and displays limited quantification limits [8]. GC and Karl Fischer are precise techniques but are costly and require well-trained operators [9]. In this context, there is still a need to develop low-cost, simple, rapid, precise, and low-sample-consuming methods to identify and quantify water adulteration in ethanol fuel.

Several alternative methods have been proposed for water quantification in ethanol, including analytical approaches based on infrared absorption spectroscopy [10], electronic tongue [11], photothermal transparent transducer [12,13,14], acoustic signature analysis in ultrasonic signals [15], researching the difference in solubility of sodium chloride in ethanol and water [8], and fluorescence spectroscopy [3].

Electrochemical impedance spectroscopy (EIS) is a widely used technique for in-depth studies of diverse materials and electrode interfaces, including those found in applications such as dye-sensitized solar cells, supercapacitors, batteries, implantable neural electrodes, gas/liquid sensors, and related fields [16,17,18,19]. Its popularity arises from its ability to conduct non-destructive analyses. In the context of biofuel analysis, EIS has emerged as a promising analytical tool for monitoring and evaluating the biodiesel and diesel-biodiesel blends [20,21] and aging processes of biodiesel fuel [22], and for assessing corrosion of metals in bioethanol-gasoline mixtures [23]. Most of the electrochemical cells used for this purpose are based on a capacitive system, which must have a large surface area and a short distance between the parallel plates of the capacitor to provide conditions for analyzing the dielectric constant of these mixtures. Even though these challenges are surpassed, the electrochemical cell still faces the challenge of using a large volume of analyte. In this scenario, miniaturized devices with millimeter/micrometer dimensions have already been successfully applied; however, they are built using photolithography, which increases the overall cost of the device, is time-consuming, and requires a well-trained operator [24].

3D-printing technology is revolutionary in the electrochemical field [25]. Additive manufacturing is fast, low-cost, and can be used to prototype devices from the macroscale [26,27,28] to the microscale [29,30]. Here, to overcome the challenges mentioned before, we prototyped a miniaturized, low-cost, and low-sample-consuming electrochemical 3D-printed system for fast and precise quantification of ethanol content based on electrochemical impedance measurements.

## 2. Materials and Methods

### 2.1. Three-Dimensional-Printed System

**Figure 1:**
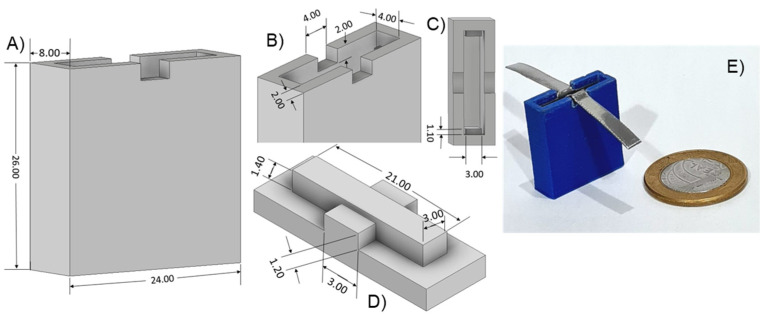
Three-dimensional-printed two-electrodes electrochemical impedance spectroscopy cell: (**A**) isometric view, (**B**,**C**) entrance, (**D**) cover, and (**E**) an image of the cell assembled with the inox electrodes and a coin to reference. All dimensions are in millimeters.

The electrochemical cell was printed using 0.1 mm polylactic acid (PLA) in a 3D-printer Sethi^®^ 3D, model S3. We modeled the cell in CAD using Autodesk Inventor^®^ 2022, sliced with Simplify 3D^®^. This miniaturized system comprised a monolithic cell and a cover with 26.0 × 24.0 × 8.0 mm overall dimensions, as shown in Figure 1A. The inner part of the cell has two electrode separators of 3.0 mm, which avoid electric short-circuiting and accommodate the two inox electrodes of 24 × 21 × 0.5 mm (Figure 1B,C). The cell contains two grooves (4.00 × 3.60 × 2.00 mm) to allow electric contact, where the cover (Figure 1D) is placed. Figure 1E shows an image of the miniaturized cell compared to a coin. The cell and cover were printed with 0.1 mm resolution, which led to 1 h and 11 min of printing time. The cell weighs only 4.13 g and costs approximately USD 0.61. Once the inox plates are in position, a capacitive system separated by 3 mm is obtained.

### 2.2. Electrochemical Measurements

We performed the analytical investigation using two ethanol sources: (1) ethanol PA (CRQ Quimica^®^, São Paulo, Brazil, 99.5% purity) and (2) ethanol fuel as bought in the gas station (bioethanol). The blends consist of DI water from Direct-Q 3 UV Merck Millipore in the ethanol source, ranging from 0 to 100% in 10% intervals, (*w*/*w*) to ethanol PA, and (*v*/*v*) to ethanol fuel. We used Gay-Lussac and Cartier alcohol densimeters to determine all water/ethanol blends before the electrochemical analysis and a DMA 4500 M density meter to determine all gas stations’ ethanol.

**Figure 2:**
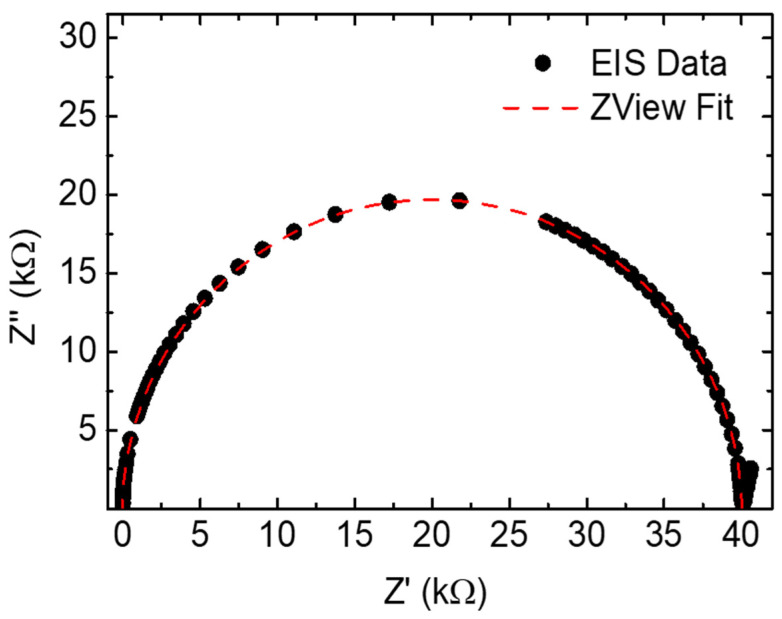
Nyquist plot of a typical dataset and its fit represented by the RC equivalent circuit.

All electrical/electrochemical measurements were performed using 1 mL of the sample using a Hewlett Packard 4192A impedance meter. The frequency sweep was from 5 Hz to 13 MHz with an amplitude of 1 V and BIAS 0 V. For each blend concentration, 6 repetitions of EIS measurements were made. Figure 2 shows the typical dataset and the ZView fit of the RC equivalent circuit.

The ZView software, version 3.0 from Scribner Associates^®^ (Southern Pines, CA, USA), was used for data analysis and calculations of the capacitances (C) and dielectric constants ($\varepsilon_{r}$) of the blends using Equation (1). Adjustments were made using the electrical RC equivalent circuit.

(1) $$\varepsilon_{r}=\frac{C}{C_{0}}=\frac{C}{\frac{\epsilon_{0}A}{d}}$$

where C and $C_{0}$ are capacitances with and without a dielectric between the plates, respectively. The identification of the area (A) used in the calculations was determined via the electrodeposition process, using 1 mL of copper chloride (CuCl) at 1.8 V for 1 min. A thin copper film was formed on the steel, and the effective area of 2.93 cm^2^ was obtained with ImageJ software, version 1.53k. With the area quantified, the dielectric constant can be obtained through Equation (1), where $\epsilon_{0}$ is the vacuum permittivity, and the average is calculated for each blend.

The experiment was performed to identify different ethanol solutions within a 3D-printed cell chamber as follows: 1 mL of each solution was placed in a sensing area, and the impedance was recorded from 5 Hz to 13 MHz. Before collecting new data, the chamber was washed with DI water and dried with nitrogen.

## 3. Results and Discussion

**Figure 3:**
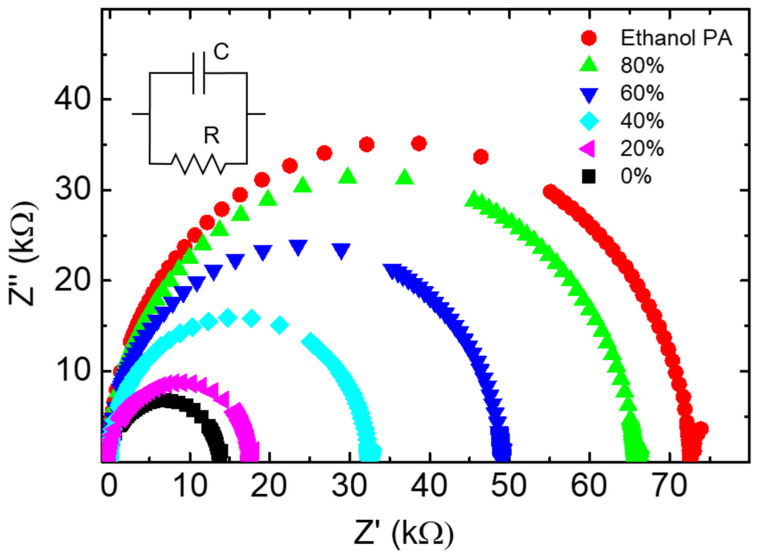
Nyquist plots for different ethanol concentrations in ethanol/water solutions.

Figure 3 shows that the arc diameter of the Nyquist plots decreases with the increase in water concentration in the solution, reaching the maximum value for the ethanol PA (red dots) and the minimum with DI water (black squares). Therefore, using the 3D cell configuration clearly distinguishes ethanol concentrations in the water/ethanol solutions.

The impedance data analysis considers the elemental equivalent circuit of the printed cell, the RC circuit. We fitted the equivalent circuit to the recorded impedance data to obtain the capacitance for each ethanol concentration in the solutions. The dielectric constants were calculated from the values of capacitance obtained using Equation (1). The fitting EIS parameters to ethanol PA grade and ethanol fuel are listed in Table 1.

**Figure 4:**
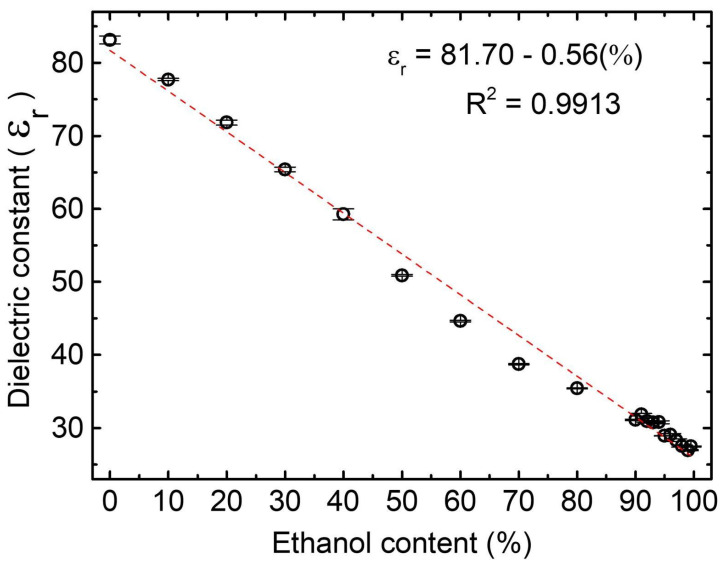
Dielectric constant/Ethanol content from ethanol PA mixture.

The analysis involves solutions with ethanol concentrations ranging from 0% to 100%. At 0% concentration, the solution contains only water, while at 100%, it contains pure ethanol of PA grade. The linear relation of ethanol content and dielectric constant is shown in Figure 4. The curve fitting makes it possible to predict the percentage of ethanol based on the dielectric constant analyzed in our 3D cell. The slope of the curve obtained for PA ethanol was −0.558 ± 0.012 with an R2 value of 0.9913. The sensibility is obtained through the slope module of the curve, then 0.55 of sensibility for the PA ethanol.

**Figure 5:**
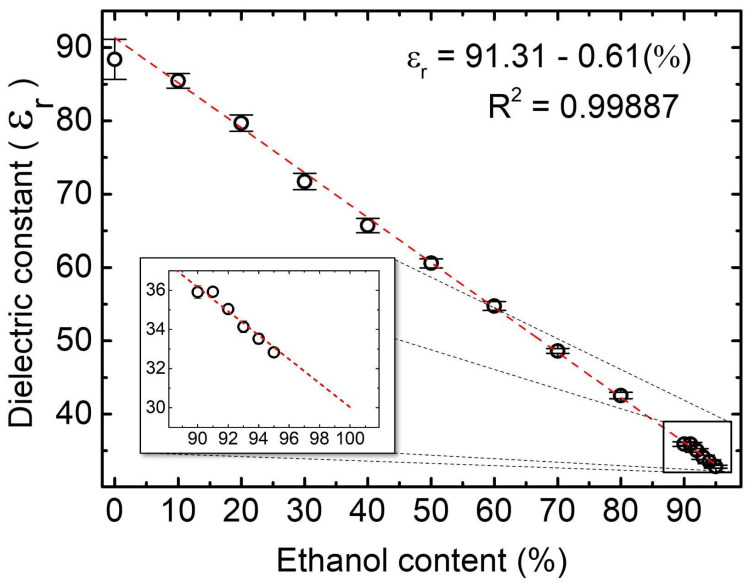
Dielectric constant/Ethanol content from the gas station mixture. The inset highlights the curve in the 90 to 100% range.

The experiment was also performed with fuel ethanol from a gas station. The ethanol/water blends were then subjected to a digital densimeter and impedance spectroscopy, resulting in a linear relationship (Figure 5). The analysis involves solutions with bioethanol concentrations ranging from 0% to 95%. The slope of the curve obtained for the gas station ethanol was −0.612 ± 0.006 with a 0.998 R2 value. Although the linear behavior remains the same, the slope has increased compared to Figure 4. This change in slope could be attributed to other compounds diluted in fuel ethanol, such as additives and dyes. The inset in Figure 5 shows that it is possible to extrapolate the results in the most important range of analysis, between 94 and 100%, with a reliability of 0.998 for bioethanol content.

Four ethanol fuel samples (A, B, C, and D) were taken from different gas stations. Their ethanol content was estimated using the proposed system, using the calibration curve obtained for the gas ethanol fuel ($\varepsilon_{r}=91.310-0.612\;Ethanol\%$), and compared with the results obtained from the digital densimeter (Table 2).

Table 1 shows that the percentage of ethanol content estimated using the proposed system with the 3D-printed cell is close to the content displayed by the digital densimeter. In addition, the proposed method has all the requirements to quantify the presence of an upper limit of water (4.5%, *v*/*v*) in a commercial sample of HE, as requested by governmental legislation, through the analysis of a small volume of the sample in a precise, cheap, and fast manner when compared to the conventional methods (Karl Fischer, chromatography, and digital densimeter).

## 4. Conclusions

We demonstrate that the electrochemical impedance spectroscopy (EIS) method collected in a miniaturized 3D-printed cell can quantify ethanol and ethanol fuel mixtures with water via the dielectric constant of the blends. In addition to designing the device and method, we compared the ethanol content of samples from four different gas stations using our system to the value obtained with a digital densimeter, and all values matched.

The [16,17] miniaturized EIS cell is monolithic, with electrode separators made of insulating thermoplastic integrated into the system. At the same time, the only mobile part is the cover, making it easy to assemble and handle. This portable (~26 × 24 mm) miniaturized cell has a maximum capacity of ~1 mL, suitable for lab-on-a-chip and portable analysis. The whole 3D-printed EIS two-electrodes cell costs less than USD 1.00, including the inox electrodes, and each unit can be printed and assembled in less than two hours. The cell is very stable, but the PLA may face chemical instabilities in the long term, which is intrinsic to the material. Although low-cost, the PLA cell can be sustainably recycled since the plastic can be turned into pellets for future extrusion to become a filament. The inox electrodes can be polished until mirror-finish to sustain electrochemical features. This system may be further coupled to a mini-potentiostat interfaced with a smartphone for in loco electroanalysis.

## Figures and Tables

**Table 1:** The fitting parameters for the EIS ethanol content in ethanol PA grade and ethanol fuel calibration curve and the digital densimeter result for each blend.

Ethanol Content (%)	Parameters					
	Ethanol PA			Ethanol Fuel		
	R (kΩ)	C (10^−11^ F)	ε_r_	R (kΩ)	C (10^−11^ F)	ε_r_
0	13.307	7.187	83.143	10.580	7.641	88.392
10	15.905	6.718	77.714	13.678	7.377	85.341
20	18.917	6.208	71.818	17.968	6.887	79.668
30	25.836	5.653	65.393	32.248	6.201	71.728
40	29.449	5.122	59.246	34.476	5.681	65.720
50	39.751	4.399	50.883	38.339	5.236	60.573
60	48.534	3.858	44.633	43.371	4.733	54.747
70	58.689	3.349	38.741	40.732	4.204	48.628
80	66.018	3.062	35.425	41.448	3.678	42.541
90	71.657	2.691	31.127	46.321	3.104	35.909
91	73.139	2.751	31.829	44.617	3.105	35.923
92	68.250	2.674	30.927	46.736	3.029	35.041
93	75.371	2.659	30.759	49.316	2.951	34.137
94	66.519	2.661	30.786	50.594	2.898	33.526
95	71.872	2.501	28.933	50.792	2.838	32.830
96	78.086	2.513	29.069	-	-	-
97	79.996	2.447	28.311	-	-	-
98	78.810	2.379	27.525	-	-	-
99	77.629	2.331	26.969	-	-	-
99.5	71.753	2.375	27.471	-	-	-

**Table 2:** Ethanol content in ethanol fuel from different gas stations analyzed using EIS and digital densimeter.

Gas Station	Dielectric Constant ± 0.30	EIS Ethanol Content (%) ± 0.30	Digital Densimeter Ethanol Content (%) ± 0.16
A	33.12	95.08	95.26
B	32.94	95.38	95.24
C	32.50	96.09	95.31
D	32.73	95.72	95.23

## Data Availability

Data are contained within the communication.
